# Retrospective Study of Osteopathic Manipulative Treatment on Length of Stay and Opioid Use After Vestibular Schwannoma Resection

**DOI:** 10.1097/ONO.0000000000000077

**Published:** 2025-08-14

**Authors:** Alice I. Chen, Shahrokh Golshan, Marc S. Schwartz, Rick A. Friedman

**Affiliations:** 1 Department of Family Medicine, Center for Integrative Medicine, UC San Diego School of Medicine, La Jolla, California; 2 Department of Psychiatry, UC San Diego School of Medicine, La Jolla, California; 3 Krupp Center for Integrative Research, Center for Integrative Health, UC San Diego, La Jolla, California; 4 Department of Neurosurgery, UC San Diego School of Medicine, La Jolla, California; 5 Department of Otolaryngology, UC San Diego School of Medicine, La Jolla, California

**Keywords:** Length of stay, Opioid use, Osteopathic manipulative treatment, Postoperative pain, Vestibular schwannoma

## Abstract

**Objective:**

This study aimed to evaluate the effect of integrating osteopathic manipulative treatment (OMT) in the acute postsurgical care of patients after vestibular schwannoma resection.

**Methods:**

This retrospective observational cohort study took place at a single tertiary care academic health center with a high-volume vestibular schwannoma program. Adults 18 years or older who underwent primary microsurgical resection of a vestibular schwannoma between November 2017 and October 2021 were included in the study. Patients were grouped by exposure to postoperative OMT, defined as receiving at least 1 OMT session during the postresection admission. The primary outcome measure was total inpatient length of stay (days), and the secondary outcome measures were daily and total opioid use (morphine milligram equivalents).

**Results:**

There were 502 patients included in this study, 284 (57%) received OMT during their postresection hospitalization. The 2 groups were similar in demographics (except race, *P* = 0.03), health history, tumor size, surgical approaches, and postoperative course. Patients who received postoperative OMT had a significantly shorter hospital length of stay compared to patients who did not receive OMT (OMT: 3.42 ± 1.41 days, 95% confidence interval [CI]: 3.26-3.59; no OMT: 3.96 ± 2.35 days, 95% CI: 3.65-4.28; F_(1,501)_ = 10.29, *P* = 0.001). The OMT group had a significantly lower daily opioid consumption over time (F_(1,1382)_ = 31.00, *P* < 0.001).

**Conclusions:**

Receiving OMT after vestibular schwannoma resection is associated with a shorter length of stay and less daily opioid use.

This is a retrospective cohort study using routinely collected patient data to examine the effect of osteopathic manipulative treatment (OMT) on patients after vestibular schwannoma (VS) resection surgery. Given the availability of an inpatient osteopathic manipulative medicine (OMM) consultation service at this high-volume VS program, our research aims are to determine the impact of integrating OMT into routine postresection care on hospitalization length and quantity of opioid medication consumed. We hypothesize that implementation of postoperative OMT will result in a significant reduction in both hospitalization length and opioid use.

## BACKGROUND/RATIONALE

As healthcare spending rises in the United States (US), value-based healthcare has come to the forefront of conversations. Among this effort is encouragement of interdisciplinary collaboration to promote recovery after illness (1). While studies have identified general debility, frailty, and preoperative mobility as factors that may contribute to longer LOS, there are few treatment options beyond physical and occupational therapy readily available to address these patient factors (2–4).

Additionally, despite postcraniotomy pain being more common in surgeries involving the skull base compared to other intracranial locations (5,6), there is mixed-quality evidence on the efficacy of opioid and nonopioid analgesic options and no consensus recommendations currently exist (7–9).

OMM is a noninvasive health-oriented approach to patient care that involves structural diagnosis and manual treatment (10). Osteopathic principles emphasize the interrelationship between the body’s structure and function and postulates that addressing “somatic dysfunction”—defined as impaired or altered physiologic or mechanical capabilities of the body—restores the body’s inherent ability to heal and recover (11). These somatic findings are an indication for “OMT.” OMT includes a variety of different manual techniques and approaches (12).

Growing literature demonstrates OMT as having potential benefits in other hospitalized populations. OMT may reduce pain, improve respiratory capacity, and shorten LOS after cardiac surgery (13,14) and improve cardiac function and perfusion in patients intubated and sedated after coronary artery bypass surgery (15). A pilot study showed promise in OMT for pain management and physical debility after lumbar microdiscectomy (16) and in decreasing pain, morbidity, and LOS after major abdominal surgery (17). OMT has been documented to be safe in multiple clinical scenarios (18). While the mechanism of OMT is not fully known, there are anatomic relationships impacted by VS and their resection surgeries that OMT may influence, including the trigeminocervical complex (19), the myodural bridge (20–22), the vestibulocollic reflex (23,24), and the glymphatic system (25,26).

No studies have evaluated the role of OMM or OMT in the perioperative management of patients with VS.

## MATERIALS AND METHODS

### Study Design

This is a retrospective observational cohort study comparing 2 groups across 1 surgical event and the associated days of hospitalization.

### Setting

This study included patients who underwent microsurgical resection of VS at a single tertiary care academic health center, UC San Diego Health, between November 2017 and October 2021. All patients were operated on by 1 neurotologist and 1 neurosurgeon with decades of experience in VS care (authors R.A.F vs R.F and M.S.S vs M.S.).

### Patients

All records of adult patients greater than 18 years of age who underwent microsurgical VS resection at this institution who had a valid primary diagnosis of VS (ICD-10 D33.3) and a Current Procedural Terminology code for craniotomy approaches used at this institution (61520, 61526, 61530, 61591, 61595, and 61596) were included in the study. Patients were excluded if they were undergoing revision surgery, if revision surgery data were missing, or if they were missing primary and secondary outcome measures data (hospital LOS and opioid medication used measured in morphine milligram equivalent [MME]). No other inclusion or exclusion criteria were used. Institutional Review Board approval was obtained (180978XL). The STROBE guideline is used for reporting (27).

### Variables

The primary outcome measure was total hospital LOS (days), and the secondary outcome measures were daily and total opioid medication use (MMEs) during the postresection hospitalization.

Total hospital LOS is a common proxy for measuring functional status and progress in enhanced recovery after surgery protocols (28). It was selected as a measure of OMT effect, with the premise that a shorter LOS may be a result of quicker time to achieving discharge milestones (29). Hospital length of stay (LOS) was calculated by the clinical informatics specialist as the difference between time of admission and time of discharge down to the minute prior to the data analysis.

Daily and total opioid medication use were also selected as a measure of OMT effect. They are routinely recorded, with doses easily converted to a quantifiable universal unit. As nociceptive pain after surgery is expected to some degree, daily opioid consumption over time was selected to better capture nuanced effects of OMT on pain and recovery over the duration of the hospital stay. Total opioid consumption per participant is the sum of daily opioid use consumed after the resection surgery. Each hospital day was based on the 24 hours of that date. As such, total opioid use per participant may be a function of their LOS. Both are limited in that they do not capture trends of opioid use after discharge. Total opioid consumption was calculated by the clinical informatics specialist before the data analysis.

OMT services were introduced at this hospital in March 2019. Patients who had surgeries before this date were classified into the no OMT group (n = 145). At the start of this institution’s inpatient OMM program, the head and neck surgery team began placing OMM consultation orders at the time the patient was downgraded from intensive care unit level care to the neurosurgical step-down unit, routinely on postoperative day 1. Osteopathic care took place on the same day the orders were placed, unless that day fell on a weekend, holiday, or when consultation services were unavailable. In those cases, OMT was provided on the next available date. There were 357 patients who had VS resection surgery from March 19, 2019 to October 28, 2021 who met inclusion criteria, of which 284 (80%) received OMT and were classified into the OMT group. Sixty-five patients did not receive OMT because of administrative reasons (ie, no OMM consultant available), 4 did not have a reason documented, and 4 patients declined OMT (ie, 2 reported no pain, 1 declined due to lumbar drain, and 1 was not interested). These patients (n = 73) were all included in the no OMT cohort (n = 218).

Osteopathic consultation included a relevant history, physical exam, and a standard osteopathic structural evaluation followed by OMT based on those findings. Aligned with standard osteopathic medical practice, the structural evaluation aimed to diagnose somatic dysfunction through a comprehensive and precise manual assessment of the entire body (including the cranium, the entire spine and sacrum, pelvis, abdomen, ribcage, upper and lower extremities). Somatic dysfunction was identified using the standard osteopathic TART criteria (defined as presence of tissue texture alterations, asymmetry of bilateral musculoskeletal structures, range of motion differences in joints, and tenderness of regions to palpation) (12). The osteopathic treatment approach was aligned with standard osteopathic medical practice in the acute care setting (30) and techniques used included balanced ligamentous tension/balanced membranous tension (31) and myofascial release (30). These techniques involve light touch and gentle positioning. They do not involve engagement of restrictive or anatomic barriers, nor do they utilize any direct pressure or force to mobilize tissue or bony structures.

The history and evaluation ranged from 10 to 20 minutes, and the treatment duration ranged from 20 to 45 minutes. Follow-up OMT was provided on consecutive days unless interrupted by weekends, holidays, or consultant availability. The osteopathic evaluation and OMT were performed by a physician board-certified in Osteopathic Neuromusculoskeletal Medicine with training in treating acute postoperative patients, and all but 3 patients received OMT from the same OMM attending (author A.C.).

### Data Sources/Measurement

All clinical data were collected during the hospitalization, before the study initiation, and all staff were blinded to the study’s aims at the time of data collection. All data were captured after discharge from the hospital. Data accuracy of manually extracted data was performed by the first and second authors of this study by spot checking during the data cleaning phase.

All outcome measures—total hospital LOS and daily and total MME doses—were continuous variables and extracted by a clinical informatics specialist. Age, sex, and body mass index (BMI) were continuous variables, while race, ethnicity, smoking history, and surgical approach were categorical variables. For race, values that fell below 5 were grouped into the “other” category. Return to the operating room and readmission within 30 days were binary variables and captured by the presence of an operative report within the surgical admission and an admission note within 30 days of the discharge date.

Diagnosis of hypertension, diabetes, and neurofibromatosis 2 were captured by the presence of diagnosis codes in the medical records (yes/no). Tumor size was analyzed as a continuous variable and captured from the operative reports, and if not available there, the neurosurgeon progress notes from the presurgery consultation were used. Postoperative complication data were captured through manual chart review (yes/no).

Exposure to OMT intervention and its frequency were determined based on the presence of a time stamp of the OMM consultation order and an associated completed progress note and were confirmed through manual chart review. Unless otherwise stated above, a clinical informatics specialist extracted all data.

### Bias

All patients were routinely referred to OMM on postoperative day one. Details on referral processes and percentages of patients who received OMM consultations are reported in the variables section. Additionally, groups were compared on possible confounding variables, such as tumor size and rates of postoperative complications.

### Study Size

The study size was based on the number of cases that took place during the study period, as detailed in the *Patient*s’ section above.

### Statistical Methods

Descriptive statistics and exploratory graphing were used to assess the distribution, normality, and homogeneity of the data. Continuous outcome data were transformed as needed. Groups were compared using analyses of variance and Chi-square tests (or Fisher exact tests) for outcomes measured at 1 time point (total LOS and total MME use) and linear mixed effect models with 2 grouping factors (OMT, no OMT) and up to 15 hospital days for MME use per day of hospitalization. Pearson Correlation was used to assess relationships between selected outcomes. Furthermore, if applicable, nonparametric tests were used to confirm the main findings. All statistical tests were conducted using SPSS version 29 using 2-tailed tests with a *P* value of 0.05 or less indicating statistical significance.

## RESULTS

### Patients

A total of 511 records met selection criteria and were extracted and examined for eligibility. Nine patients were excluded due to having a revision surgery (n = 2) or due to missing revision surgery data (n = 7). Five hundred two patients were included in the final analysis. Of the 502 patients, 284 (56.6%) were classified into the OMT group and 218 (43.4%) were classified into the no OMT group.

### Descriptive Data

There were no significant differences in baseline demographics (age, BMI, biological sex, and ethnicity), health history (smoking history/status, type 2 diabetes, hypertension, neurofibromatosis 2, or receipt of prior radiation therapy for the tumor), or tumor size between the 2 groups (Table 1). Race distribution was statistically different between groups (*P* = 0.03), with the no OMT group having a higher number of Asian Americans. Three patients from the OMT group (0.6%) had missing data for ethnicity, hypertension history, and diabetes history, and 6 patients were missing tumor size (OMT: 5 [1.0%], control: 1 [0.2%]). Additionally, both groups exhibited similar distributions regarding surgical approaches, with the most common surgical approach in both groups being the translabyrinthine craniotomy. Rates of postoperative complication, unplanned returns to the operating room within that same hospitalization, and readmissions to the hospital within 30 days were similar between groups. There were no mortalities in this dataset.

**TABLE 1.** Cohort demographics, medical history, tumor size, surgery details, and postoperative events

	No OMT (n = 218)		OMT (n = 284)		Total (n = 502)		*P* value
	Mean/n	S.D./%	Mean/n	S.D./%	Mean/n	S.D./%	
Demographics							
Age (years)	48.1	12.7	48.8	12.3	48.5	12.4	0.50
Body mass index	27.0	6.0	27.3	5.9	27.2	5.9	0.55
Sex^a^							
Female	88	40.4%	99	34.9%	187	37.3%	0.21
Male	130	59.6%	185	65.1%	315	62.7%	
Race^a^							
White	160	73.4%	226	79.6%	386	76.9%	0.03
Asian	23	10.7%	13	4.6%	36	7.2%	
Other	35	16.1%	45	15.8%	80	15.9%	
Ethnicity^a^							
Hispanic	22	10.1%	22	7.8%	44	8.8%	0.38
Non-Hispanic	196	89.9%	259	92.2%	455	91.2%	
Health history							
Neurofibromatosis 2^b^	6	2.8%	3	1.1%	9	1.8%	0.19
Prior radiation therapy for VS^a^	9	4.1%	16	5.6%	25	5.0%	0.44
Hypertension^a^	42	19.3%	48	17.1%	90	18.0%	0.53
Diabetes^a^	9	4.1%	12	4.3%	21	4.2	0.94
Smoking history^a^							
Never	174	79.8%	229	80.6%	403	80.3%	0.91
Former	9	4.1%	13	4.6%	22	4.4%	
Active	35	16.1%	42	14.8%	77	15.3%	
Surgical features							
Tumor size (mm)	21.1	11.2	19.5	9.8	20.2	10.5	0.10
Surgical approach^a^							
Middle Fossa	48	22.0%	79	27.8%	127	25.3%	0.31
Translabyrinthine	116	53.2%	132	46.5%	248	49.4%	
Retrosigmoid	54	24.8%	72	25.4%	126	25.1%	
Combined	0	0.0%	1	0.4%	1	0.2%	
Postoperative course^a^							
Any complication^a^	45	20.6%	43	15.1%	88	17.5%	0.11
Cerebral edema^b^	4	1.8%	5	1.8%	9	1.8%	1.00
Cerebrospinal fluid leak^a^	26	11.9%	24	8.5%	50	10.0%	0.20
Cerebrovascular accident^b^	2	0.9%	0	0.0%	2	0.4%	0.19
Dural sinus thrombosis^b^	5	2.3%	2	0.7%	7	1.4%	0.25
Infection^b^	5	2.3%	1	0.4%	6	1.2%	0.09
Intracranial bleed^a^	6	2.8%	7	2.5%	13	2.6%	0.84
Pulmonary embolism, deep vein thrombosis^a^	7	3.2%	5	1.8%	12	2.4%	0.29
Seizure^b^	2	0.9%	1	0.4%	3	0.6%	0.58
Unplanned return to OR^a^	6	2.8%	6	2.1%	12	2.4%	0.59
Readmission within 30 days^a^	19	8.7%	21	7.4%	40	8.0%	0.59
Mortality	0	0.0%	0	0.0%	0	0.0%	NA

Data are presented as mean (SD) or N (% of total).

*P* value from ANOVA or

a Chi-square test or

b 2-sided Fisher’s exact test if small sample size.

ANOVA indicates analyses of variance; NA, not available.

### Outcome Data

The intervention group received on average 2 OMT sessions (range: 1–7), with 29% of patients (n = 82) receiving 1 treatment, 43% (n = 123) receiving 2 treatments, 26% (n = 74) receiving 3 treatments, 1.8% (n = 4) receiving 4 treatments and 0.4% (n = 1) receiving 7 treatments. There was a significant correlation between the total LOS and total number of OMT sessions (Pearson correlation: 0.47, *P* < 0.001). Two hundred fifty (88%) patients received their first OMT session on postoperative day 1, 19 (7%) on postoperative day 2, and 15 (5%) on postoperative day 3 or later. Patients received no more than 1 OMT per day.

### Main Results

The average LOS for the entire dataset was 3.66 ± 1.90 days with 73.7% of patients discharged by the end of hospital day 3, 13% by the end of hospital day 4 (total of 86.7%), and 4.7% by the end of day 5 (total of 91.4%). Patients receiving OMT had a significantly shorter hospital stay compared to those who did not (OMT: 3.42 ± 1.41 days, 95% confidence interval [CI]: 3.26-3.59; no OMT: 3.96 ± 2.35 days, 95% CI: 3.65-4.28, F_(1,501)_ = 10.29, *P* = 0.001, eta square = 0.02). The OMT group was significantly more likely to be discharged by the end of hospital day 2 compared to the no OMT group (37.0%, 25.7%; *P* < 0.001). (Table 2).

**TABLE 2.** Number of participants discharged per day in each group

Discharge day	No OMT (n = 218)		OMT (n = 284)		Total (n = 502)		Cumulative %
	n	%	n	%	n	%	
1	1	0.5%	0	0%	1	0.2%	0.2%
2	56	25.7%	105	37.0%	161	32.1%	32.3%
3	97	44.5%	111	39.1%	208	41.4%	73.7%
4	28	12.8%	37	13.0%	65	13.0%	86.7%
5	11	5.1%	13	4.6%	24	4.8%	91.4%
6	7	3.2%	8	2.8%	15	3.8%	94.4%
7	3	1.4%	6	2.1%	9	1.8%	96.2%
8	5	2.3%	2	0.7%	7	1.4%	97.6%
9	2	0.9%	0	0	2	0.4%	98.0%
10	1	0.5%	0	0	1	0.2%	98.2%
11	1	0.5%	1	0.4%	2	0.4%	98.6%
12	2	0.9%	1	0.4%	2	0.4%	99.0%
13	2	0.9%	1	0.4%	3	0.6%	99.6%
14	1	0.5%	0	0	1	0.2%	99.8%
15+	1	0.5%	0	0	1	0.2%	100.0%

n denotes the number of participants admitted on that day and % is of total in that group. Cumulative % refers to total participants in study.

There was no significant difference between the 2 groups in their total opioid use (86.79 ± 88.71 MME for the OMT group; 98.15 ± 133.58 MME for the no OMT group), more likely due to the impact of the total hospitalization length on this computation. Although the daily MME use on day 1 was slightly higher for the OMT group (42.25 ± 33.76 MME; no OMT group: 40.12 ± 36.59 MME), the OMT group reduced medication use significantly more rapidly than the no OMT group (group by days: F_(1,1382)_ = 31.00, *P* < 0.001). There were significantly fewer patients on postoperative day 3 taking opioid medication in the OMT group compared to the no OMT group (63.0%, 73.9%, χ^2^ = 6.03, *P* = 0.014). There were no significant differences in the percentage of patients taking opioids between groups on days 4 and 5, and there were significant differences in these percentages on day 6 through day 9 (Table 3).

**TABLE 3.** Daily average opioid use (in morphine milligram equivalents [MME]) per hospital day of each group

Day	No OMT (n = 218)				OMT (n = 284)				Total (n = 502)			X_2_	*P* value
	n	%	Mean	S.D.	n	%	Mean	S.D.	n	Mean	S.D.		
1	218	100	40.1	36.6	284	100	42.3	33.8	502	41.3	35	NA	NS
2	218	100	23.1	26.8	284	100	21.9	25.2	502	22.4	25.9	NA	NS
3	161	73.9	20.0	27.5	179	63.0	20.3	29.9	340	20.1	28.8	6.03	0.01
4	64	29.7	26.2	41.8	68	23.9	24.9	27.3	132	25.5	34.9	1.87	0.17
5	36	16.5	31.9	54.2	31	10.9	18.7	21.4	67	25.8	42.6	3.33	0.07
6	25	11.5	29.4	60	18	6.3	18.1	22.4	43	24.7	47.9	4.14	0.04
7	18	8.3	23.8	42.6	10	3.5	16.1	17.5	28	21.1	35.5	5.25	0.02
8	15	6.9	9.0	23.2	4	1.4	9.4	11.3	19	9.1	21	10.11	<0.01
9	7	3.2	16.8	23.5	1	0.4	15	NA	8	16.6	21.8	6.43	0.01
10	5	2.3	6.0	3.4	NA	NA	NA	NA	5	6	3.4	NA	NA
11	2	0.9	37.5	10.6	NA	NA	NA	NA	2	37.5	10.6	NA	NA
12	1	0.5	52.5	NA	NA	NA	NA	NA	1	52.5	NA	NA	NA

n denotes the number of participants admitted on that day and % is of total in that group. Opioid data presented in MME units with mean and standard deviation. *P* value from Chi-square test.

NA indicates not available; NS, not significant.

To evaluate the dose effect of OMT, a mixed-effects model was used to compare daily opioid dosage (MME) over the first 3 days by number of OMT sessions (4 groups: 0, 1, 2, and 3 OMT sessions), adjusting for LOS. There was a significant relationship between the number of OMT sessions and daily MME use (2-way interaction effect: F_(11,1316)_ = 14.30, partial eta square = 0.11, *P* < 0.001).

## DISCUSSION

### Key Results

This is the first study on OMT use in the acute postoperative period following VS resection. Our findings suggest that integrating OMT after surgery may be associated with a shorter hospital LOS and lower daily opioid use over the duration of the hospitalization.

Specifically, comparison of means revealed that postoperative OMT was associated with a reduction in LOS by approximately 0.54 days (or 12.96 hours), with an eta square of 0.02 indicating a small effect size. While this reduction may have meaningful implications for patient care and hospital efficiency, quantifying its exact value remains complex. The average cost of an overnight hospital stay in the US is approximately $3132 and in California $4471 (32), translating this 0.54 day reduction into an estimated cost savings of $2414 per patient in California. However, this estimate should be interpreted with caution, as hospital costs vary by region, facility type, and reimbursement structure. Beyond direct cost savings, the broader impact of earlier bed availability warrants consideration. In hospitals operating at or near capacity, efficient resource allocation is needed to optimize patient flow and prevent delays in critical care. Regaining bed availability 12 hours sooner may have significant downstream effects, not only facilitating smoother transitions for incoming patients but also potentially minimizing delays in critical interventions and reducing emergency department overcrowding. Given projections that the US will have a hospital bed shortage by 2032 (33), further exploring these operational benefits may be worthwhile.

Patients who received OMT also had a decrease in daily opioid use over time, with the OMT group reducing medication use significantly more rapidly than the no OMT group and with fewer OMT patients taking medication on the third day than the no OMT group. This rapid decrease in opioid consumption over time suggests a potential benefit over the course of the recovery period. One limitation to keep in mind while interpreting this information is that opioid consumption after discharge was not captured in this study, and following this value during the recovery period may provide further insight into OMT’s effect on this parameter. Additionally, the MME data in this study only include the values of patients still admitted to the hospital and therefore, are biased toward increased severity. Even among those patients with longer hospitalizations, there were significantly fewer patients in the OMT group taking pain medications from day 3 onward. It is also noteworthy that the group receiving OMT demonstrated lower average total MME use accompanied by a narrower standard deviation. The narrower standard deviation in opioid use among the OMT group, consistent over the days of hospitalization (Table 3), may reflect less variability in opioid consumption, which may suggest more consistent pain management and possibly better pain control.

The significant interaction effect between the number of OMT session and opioid medication use, adjusting for LOS, suggests the importance of the combination of time and OMT in affecting patient outcomes. Further prospective clinical trials are needed to better understand the full impact of OMT dosing on these outcomes.

Postoperative pain is a multidimensional and subjective experience, and current frameworks for guiding a comprehensive pain assessment in the clinical setting are limited in capturing this complexity (34). Opioid consumption does not always accurately reflect the pain severity or recovery progress, as patients may take opioids on a scheduled basis and/or as needed to manage other symptoms that may be interrelated with their pain. Additionally, nociceptive pain after surgery is expected to some degree, and future studies should take this into consideration with longer follow-up intervals capturing opioid consumption after discharge from the hospital and measuring other aspects of a patient's postoperative pain and symptom experience.

### Strength and Limitations, and Suggestions for Future Studies

A notable strength of this study lies in the large number of patients in each cohort and the unified care provided by the same surgical, hospital, and osteopathic teams for these patients. This resulted in standard perioperative care regimens, which minimize potential confounding factors and biases that could emerge from variations in practice patterns. Additionally, eligibility and group assignments were determined based on baseline characteristics before the start of follow-up data collection to mimic the study process of a randomized trial (35).

There are a few limitations. The largely sequential, semioverlapping cohorts may contribute to systematic differences and covariates that may be driving the differences between groups. Clinician experience and training may have influenced behavior in opioid prescribing. However, standardized postoperative order sets reduce some of this variability by predetermining medication doses and frequency based on patient-reported pain levels. All opioid medications are scheduled as needed based on pain severity, and patients’ reported pain levels drive their opioid medication use. Additionally, there was no consultation between the surgical and OMM team regarding pain medication adjustments based on OMT exposure. As awareness of this program increased, referral patterns and patient complexity may have shifted overtime and contributed to outcome differences as well. We attempted to address these covariates by looking at demographics, tumor size, health history, and complication rates, of which no differences were observed between groups. Future prospective studies with randomization and blinding would better control for these variables.

Future studies could also consider other outcome measures that impact recovery and opioid use. For example, dizziness, balance issues, and vestibular concerns are well-recognized factors that influence discharge timing (36). This study did not include these symptoms as outcome measures, as these data were not routinely collected in a standardized format in patient medical records. Prospective studies that specifically assess these symptoms and incorporate longitudinal follow-up could provide valuable insights into the effects of OMT on balance and vestibular recovery. Since these symptoms were not systematically measured, it remains unclear whether OMT had a direct impact on them in this study. However, given the study design, these factors would likely have influenced both groups similarly and are unlikely to have affected the observed outcome differences.

### Interpretation

The integration of postoperative OMT following VS resection is associated with a reduction in both hospital LOS and daily opioid medication use. This suggests that OMT may be an effective adjunct treatment to incorporate into the postoperative management of patients undergoing such procedures. The reduction may suggest clinical benefits for patients and may have broad implications for hospital efficiency and workflow, resource utilization, and healthcare costs.

While these initial findings are promising, it is important to note that the study results do not prove causation. Further research such as a prospective randomized controlled study is warranted to confirm these results.

### Generalizability

Data from this study are generalizable to patients who receive VS resections, in particular at high-volume centers with OMM programs. This surgery for a rare tumor, alongside a rare inpatient OMM service, provides valid empirical data to support the further use of OMM in this patient population and potentially more broadly in other surgical settings.

## ACKNOWLEDGMENTS

The authors would like to thank Renu Sugathan for her assistance with data extraction.

## FUNDING SOURCES

There was no funding for this study. Sources of administrative support include the Krupp Center for Integrative Research, the Center for Integrative Medicine, and the Department of Family Medicine at UC San Diego.

## CONFLICT OF INTEREST STATEMENT

None declared.

## DATA AVAILABILITY STATEMENT

Dataset available upon request of the author and is subject to approval by the institution and as allowed by law.
